# Lyme neuroborreliosis in HIV-1 positive men successfully treated with oral doxycycline: a case series and literature review

**DOI:** 10.1186/1752-1947-5-465

**Published:** 2011-09-19

**Authors:** Daniel Bremell, Christer Säll, Magnus Gisslén, Lars Hagberg

**Affiliations:** 1 Institute of Biomedicine, the Sahlgrenska Academy, University of Gothenburg, Sweden; 2 Department of Infectious Diseases, Södra Älvsborgs Hospital, SE-501 82 Borås, Sweden

## Abstract

**Introduction:**

Lyme neuroborreliosis is the most common bacterial central nervous system infection in the temperate parts of the northern hemisphere. Even though human immunodeficiency virus (HIV) -1 infection is common in Lyme borreliosis endemic areas, only five cases of co-infection have previously been published. Four of these cases presented with typical Lyme neuroborreliosis symptoms such as meningoradiculitis and facial palsy, while a fifth case had more severe symptoms of encephalomyelitis. All five were treated with intravenous cephalosporins and clinical outcome was good for all but the fifth case

**Case presentations:**

We present four patients with concomitant presence of HIV-1 infection and Lyme neuroborreliosis diagnosed in Western Sweden. Patient 1 was a 60-year-old Caucasian man with radicular pain and cognitive impairment. Patient 2 was a 39-year-old Caucasian man with headaches, leg weakness, and pontine infarction. Patient 3 was a 62-year-old Caucasian man with headaches, tremor, vertigo, and normal-pressure hydrocephalus. Patient 4 was a 50-year-old Caucasian man with radicular pain and peripheral facial palsy. Patients one, two, and three all had subnormal levels of CD4 cells, indicating impaired immunity. All patients were treated with oral doxycycline with good clinical outcome and normalization of CSF pleocytosis.

**Conclusion:**

Given the low HIV-1 prevalence and medium incidence of Lyme neuroborreliosis in Western Sweden where these four cases were diagnosed, co-infection with HIV-1 and *Borrelia *is probably more common than previously thought. The three patients that were the most immunocompromised suffered from more severe and rather atypical neurological symptoms than are usually described among patients with Lyme neuroborreliosis. It is therefore important for doctors treating HIV patients to consider Lyme neuroborreliosis in a patient presenting with atypical neurological symptoms. All four patients were treated with oral doxycycline with a good outcome, further proving the efficacy of this regime.

## Introduction

Lyme neuroborreliosis (LNB) is the most common bacterial central nervous system (CNS) infection in the temperate parts of the northern hemisphere. European LNB most often presents as a painful meningoradiculoneuritis, with or without facial palsy or other cranial neuritis (Garin-Bujadoux-Bannwarth syndrome). More uncommon symptoms include deficits of other cranial nerves, myelitis and encephalitis [1].

To date, only five single cases of co-infection with human immunodeficiency virus (HIV) -1 and LNB have been published [2-6], all of whom were treated with intravenous third-generation cephalosporins. In Sweden, the recommended treatment for LNB has long been oral doxycycline. We now present a case series of four patients with HIV-1 infection that have been diagnosed with LNB and successfully treated with oral doxycycline.

## Case presentations

### Patient 1

**Figure 1:**
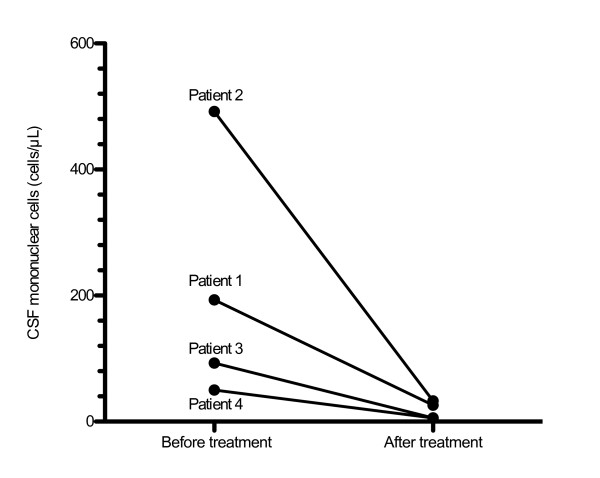
**CSF mononuclear cell count before and after treatment of Lyme neuroborreliosis with oral doxycycline**. Each line represents one patient. Mean time between CSF samplings 47 days (30-70).

This 60-year-old Caucasian man had a medical history including intermittent alcohol problems and depression, for which he was treated with disulfiram and selective serotonin re-uptake inhibitors (SSRIs). He was diagnosed with HIV 23 years earlier but did not start antiretroviral therapy (ART) until 20 years after diagnosis (lamivudine, tenofovir and ritonavir-boosted atazanavir). Two years after commencing ART, he noticed a tick bite but no erythema migrans. One year later, he was admitted to hospital with confusion, psychomotor agitation and hyponatremia (serum sodium 116 mmol/L). He had completely stopped taking his HIV medication one month earlier, after a period of deteriorating compliance. The hyponatremia was thought to have been caused by a combination of water intoxication, syndrome of inappropriate antidiuretic hormone secretion and SSRI medication. It was corrected slowly and the patient improved with regard to confusion and agitation, but he showed remaining cognitive impairment. It was also noted that he had trouble walking and suffered from radicular pain in both legs. A computed tomography (CT) scan of his brain was normal. A cerebrospinal fluid (CSF) sample four weeks after admission showed markedly elevated levels of albumin and mononuclear cells (Table 1). High CSF and serum *Borrelia *antibody titers were present and the *Borrelia *antibody index was positive, indicating intrathecal antibody production. Treatment was given with 200 mg of oral doxycycline twice daily for 10 days. At the same time, ART was re-started. Three weeks later, his pain and motor symptoms had improved. The number of CSF mononuclear cells had decreased markedly (Figure 1). At follow-up six months later, this patient's symptoms had continued to improve and the level of mononuclear cells in his CSF was down to normal (data not shown).

**Table 1:** Patients with HIV-1 and Lyme neuroborreliosis co-infection; baseline data, clinical and laboratory characteristics

**Patient no**.	Sex	Age	Years since HIV diagnosis	CD4 cell count (cells/μL)		Viral load at diagnosis of LNB (copies/mL)		Symptoms of LNB	CSF laboratory data		Borrelia diagnosis	History of tick bite
								
				nadir	at diagnosis of LNB	plasma	CSF		Mono-nuclear cells (cells/μL)	Albumin (mg/L)		
1	m	60	24	190	190	65,907	1,100,000	radicular pain, cognitive impairment	193	2790	CSF Bb-antibodies + positive Bb antibody index	yes

2	m	39	6	280	390	83,400	448,000	hearing-loss, vertigo (pontine infarction)	492	2860	CSF Bb-antibodies + positive Bb antibody index	no

3	m	62	6	180	320	< 20	219	dysgeusia, vertigo, incontinence, headache (normal pressure hydrocephalus)	93	2000	CSF Bb-antibodies + positive Bb antibody index	no

4	m	50	5	180	450	nd	nd	headache, facial palsy	50	481	CSF Bb IgG seroconversion	no

CSF: cerebrospinal fluid; m: male; nd: no data; Bb: Borrelia burgdorferi. Reference values: CD4 cells > 500 cells/μL, CSF mononuclear cells < 5 cells/μL, CSF albumin < 420 mg/L.

### Patient 2

This 39-year-old Caucasian man had primary HIV infection six years earlier. Two years after that, he fell ill with Guillain-Barré syndrome, which was treated with intravenous gammaglobulin, and ART was started with stavudine, lamivudine, saquinavir and nelfinavir given for eight months. The Guillain-Barré symptoms resolved [7]. He was admitted with slowly increasing headaches, weakness in both legs and right hand tremor. On admission, he was still without ART and had a CD4 cell count of 390 cells/μL. Two days before admission, this patient had experienced a sudden onset of vertigo and hearing loss in his right ear. A magnetic resonance imaging scan showed a pontine infarction. Levels of albumin and mononuclear cells in his CSF were markedly elevated (Table 1). *Borrelia*-antibody titers were high in both his serum and CSF, and the *Borrelia *antibody index was positive. Treatment was given with 200 mg of oral doxycycline twice daily for 19 days. His symptoms of headaches, weakness, tremor and vertigo started improving within three days of starting treatment, but the hearing loss remained. Repeated lumbar punctures showed declining levels of CSF albumin (data not shown) and mononuclear cells (Figure 1). At follow-up after six months, he was still experiencing a complete hearing loss in his right ear, but the other symptoms had subsided. He still had no ART.

### Patient 3

This 62-year-old Caucasian man was diagnosed with a primary HIV infection seven years earlier. Viral load was high and the CD4 cells were low at 180 cells/μL. ART was started with lamivudine, zidovudine and ritonavir-boosted lopinavir for one year and was re-started after four years with efavirenz, abacavir and lamivudine. One year later, the patient noted a change in the taste of coffee but no other foodstuff. In the following months, he experienced gradually increasing problems with vertigo and unsteadiness. A few months later, he also noted symptoms of tremor, urge incontinence and intermittent headaches. A CT scan revealed normal-pressure hydrocephalus. Six months later, CSF sampling showed increased levels of protein and mononuclear cells (Table 1). Culture and polymerase chain reaction tests for opportunistic infections were negative, but a cytological examination pointed to a neurotrophic infection. Four months after the first CSF analysis, a new analysis of CSF and serum revealed high titers of CSF and serum *Borrelia *antibodies and a positive antibody index, consistent with LNB. Treatment was given with 200 mg of oral doxycycline twice daily for 10 days. All the symptoms (including the change of taste of coffee) started improving within five days of treatment initiation. At follow-up two months later, the symptoms had almost completely disappeared and CSF levels of mononuclear cells and albumin had normalized (Figure 1).

### Patient 4

This 50-year-old Caucasian man had been diagnosed with HIV five years previously. The date of infection was not known. He had been treated with ART for four years, initially lamivudine, zidovudine and ritonavir-boosted lopinavir, which were subsequently changed to efavirenz, emtricitabine and tenofovir. After treatment, his CD4 cell count had risen from 180 to 450 cells/μL. He fell ill with fever and headaches, plus a rash, which was later diagnosed as erythema migrans. Some days later he noted a right-sided facial palsy. His CSF levels of albumin and mononuclear cells were elevated (Table 1). Titers of *Borrelia *immunoglobulin M (IgM) antibodies in his serum and CSF were elevated, but IgG antibodies were not. Treatment was given with 100 mg of oral doxycycline twice daily for 21 days. The symptoms improved within a few days after treatment initiation. At follow-up two months later, the patient was still experiencing a slight sensibility disturbance from the right side of his face, but all the other symptoms had subsided and CSF pleocytosis and albumin concentration had normalized (Figure 1). *Borrelia *IgG antibodies in his serum and CSF were now positive.

## Discussion

Only a few reports of HIV-1 and LNB co-infection have been presented. Previously, this was considered to be due to the non-overlapping epidemiology of the two diseases; with LNB mainly being a rural disease, while HIV-1 is more common in urban settings [5]. HIV-1 patients today who are treated with ART have a life expectancy approaching that of the general population and they seldom have opportunistic infections [8], thereby enabling them to lead active lives with outdoor activities that increase the risk of contracting *Borrelia *infection. The incidence of LNB is highest in Central Europe [9], where the prevalence of HIV-1 infection is also substantially higher than in Sweden, where these four patients were diagnosed. It can therefore be expected that HIV-1 and LNB co-infection is more common than has previously been described.

The diagnosis of LNB rests on a combination of clinical symptoms, CSF analyses and serology. The results are sometimes contradictory or difficult to interpret, making the diagnosis uncertain. In these four patients, however, the diagnosis of LNB is considered definite. At the time of diagnosis, all these patients had CSF mononuclear pleocytosis, with cell counts higher than the levels normally observed in HIV patients [10]. Patients 1, 2 and 3 all had high titers of IgG antibodies to *Borrelia *in their CSF and serum and a positive *Borrelia *antibody index, indicating the intrathecal production of specific *Borrelia *antibodies. Patient 4 seroconverted from negative to positive IgG antibodies to *Borrelia *in his serum and CSF. Furthermore, all the patients displayed a rapid response to anti-*Borrelia *treatment. There is a well-known cross-reactivity between the *Borrelia spirochete *and the *Treponema spirochete *and HIV patients are over-represented among patients with syphilis. However, screening tests for syphilis were negative in all four patients. Other bacterial, viral and fungal CNS infections were also ruled out.

Four of the five previously published cases of HIV-1 and LNB co-infection presented with typical symptoms of LNB, including bilateral facial palsy [5], headache [4], meningoradiculitis [3], and facial palsy and meningoradiculitis [2]. These four patients showed complete recovery on treatment with intravenous third-generation cephalosporins. The fifth patient presented with more severe symptoms consistent with encephalomyelitis: altered gait and difficulties in using her hands. Treatment with intravenous third-generation cephalosporins resulted in only partial recovery [6].

Three of the four patients described in this article presented with more severe, atypical symptoms and pathology than are usually seen in patients with LNB, including one with cognitive impairment, one with a pontine infarction and one with normal-pressure hydrocephalus. However, concomitant medical disorders such as alcohol abuse in Patient 1 and previous Guillain-Barré in Patient 2 might have influenced the clinical course of LNB. The atypical clinical picture might also have been caused by the long disease duration before *Borrelia *diagnosis and subsequent treatment; a couple of months for Patient 2, and more than a year for Patient 3. An explanation for the delayed diagnosis could be that more common HIV-associated opportunistic infections and other diseases were initially suspected. Apart from the concomitant medical disorders and the long disease duration, it must, however, also be suspected that these patients' impaired immunity contributed to the severity of the disease, as none of these three patients had normal levels of CD4 cells. The exact mechanisms by which impaired immunity in HIV infection might influence the course of disease in LNB remain to be clarified. Acute cerebral infarction is a known but very rare manifestation of LNB, with the pathological mechanism suspected to be a selective inflammatory process of small cerebral arteries. Patient 2 matches those previously described with the involvement of the posterior circulation and a generally favorable outcome after treatment [11]. Normal-pressure hydrocephalus in patients with LNB is an even rarer manifestation, with only a few known cases. The pathological background is not understood. As with Patient 3, previously described cases have also shown complete improvement after antibiotic treatment, with no need for ventricular shunting therapy [12].

The European Federation of Neurological Societies has published guidelines on the management of LNB [13]. According to these guidelines, patients with early LNB with CNS symptoms or patients with late (more than six months of symptoms) LNB should be treated with intravenous ceftriaxone. Of the four patients presented here, three had late LNB with CNS symptoms and were also the most immunocompromised. The good outcome of treatment with oral doxycycline in these patients, in combination with the CSF follow-up analyses, suggests that oral doxycycline is an excellent alternative to intravenous ceftriaxone in this patient group.

One interesting observation in Patients 1, 2 and 3 was the relatively higher HIV viral load in CSF compared with plasma at time of diagnosis of the *Borrelia *infection (Table 1). This shows that concomitant meningeal inflammation and the recruitment of lymphocytes to the CNS in HIV infection increase the CNS viral load, probably by the Trojan horse pathway [14]. Similar findings have been observed in patients with cryptococcal and tuberculous meningitis [15].

## Conclusions

In this case series, we present four patients with HIV-1 and LNB co-infection diagnosed in Western Sweden, an area with a low HIV-1 prevalence and a medium incidence of LNB. Thus, co-infection with HIV-1 and LNB is probably more common than previously thought. The three patients that were the most immunocompromised suffered from more severe and atypical neurological symptoms than are usually described among patients with LNB. It is therefore important for doctors treating HIV patients to consider LNB if a patient presents with neurological symptoms. All four patients were treated with oral doxycycline with a good outcome further proving the efficacy of this regime.

## Consent

Patient 1 had died at the time of writing of this article. Written informed consent was obtained for publication of this case series from the patient's brother. For Patients 2, 3 and 4 written informed consent was obtained. Copies of the written consents are available for review by the Editor-in-Chief of this journal.

## Competing interests

The authors declare that they have no competing interests.

## Authors' contributions

All the authors contributed to the design and data analysis of the study, the writing of the article and approved the final version.
